# A simplified and defined serum-free medium for cultivating fat across species

**DOI:** 10.1016/j.isci.2022.105822

**Published:** 2022-12-17

**Authors:** Rada Mitić, Federica Cantoni, Christoph S. Börlin, Mark J. Post, Laura Jackisch

**Affiliations:** 1 Mosa Meat B.V., Maastricht, Limburg 6229 PM, the Netherlands; 2 Department of Physiology, Maastricht University, Maastricht, Limburg 6211 LK, the Netherlands

**Keywords:** Biological sciences, Cell biology, Food science

## Abstract

Cultivated meat is a promising technology with the potential to mitigate the ethical and environmental issues associated with traditional meat. Fat plays a key role in the meat flavor; therefore, development of suitable adipogenic protocols for livestock is essential. The traditional adipogenic cocktail containing IBMX, dexamethasone, insulin and rosiglitazone is not food-compatible. Here, we demonstrate that of the four inducers only insulin and rosiglitazone are necessary in both serum-free (DMAD) and serum-containing media, with DMAD outperforming FBS. Two glucocorticoid receptor activators, progesterone and hydrocortisone, found in DMAD and FBS, affect differentiation homogeneity, without playing an essential role in activating adipogenic genes. Importantly, this protocol leads to mature adipocytes in 3D culture. This was demonstrated in both media types and in four species: ruminant and monogastric. We therefore propose a simplified one-step adipogenic protocol which, given the replacement of rosiglitazone by a food-compatible PPARγ agonist, is suitable for making cultivated fat.

## Highlights

• Serum-free medium outperforms FBS-containing medium for differentiation
• Only two inducers, rosiglitazone and insulin, are needed for serum-free adipogenesis
• The 1-step protocol, instead of the traditional 3-step, can be used in both 2D and 3D
• The simplified protocol translates across several species (cow, sheep, pig, and mouse)

## Graphical abstract

**Figure:**
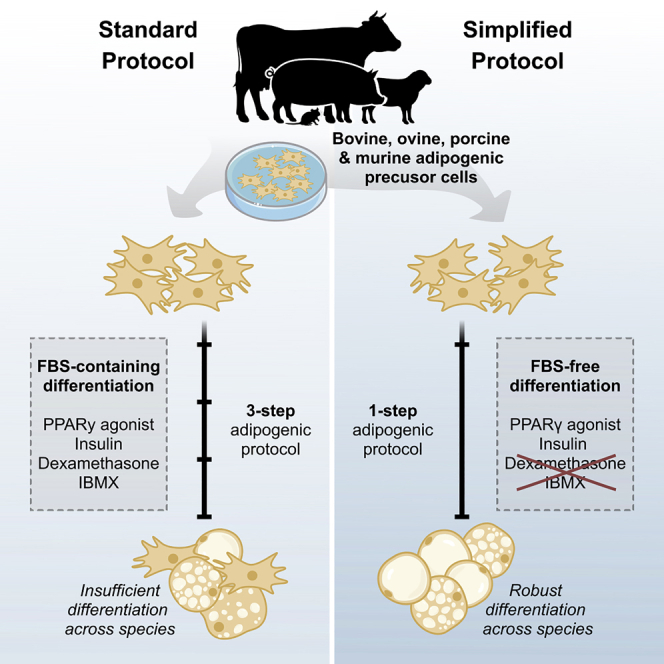

## Introduction

Intensive animal farming is one of the major contributors to global environmental degradation and poses great concerns on animal welfare.^1^ Cultivated meat is a technology that has been proposed to overcome issues brought by conventional meat production.^2^^,^^3^ The concept is based on the ability of stem cells to proliferate to significant numbers and subsequently, with appropriate stimuli, differentiate into a desired tissue.^4^ The final product should resemble the taste, texture, and nutritional value of conventional meat, and for that purpose the addition of fat tissue is necessary. Fat tissue plays an essential role in body homeostasis through its energy storing and endocrine functions.^5^^,^^6^^,^^7^^,^^8^ Adipocytes make up most of the fat tissue volume and are unique among cells because 95% of their entire cell body is composed of one large lipid droplet, containing high energy molecules: triglycerides.^9^^,^^10^ Their unique physiological properties and the lack of a robust cytoskeleton make them important for meat taste and tenderness of meat products.^11^^,^^12^^,^^13^^,^^14^ Fat tissue has been reported as the most important contributor to meat flavor across species,^15^^,^^16^^,^^17^ and fatty taste or oleogustus, has been proposed as the sixth basic taste.^18^ Given its role in palatability and nutritional value of a meat cut, developing an efficient protocol for fat production through adipogenic differentiation is of great importance.

**Figure 1:**
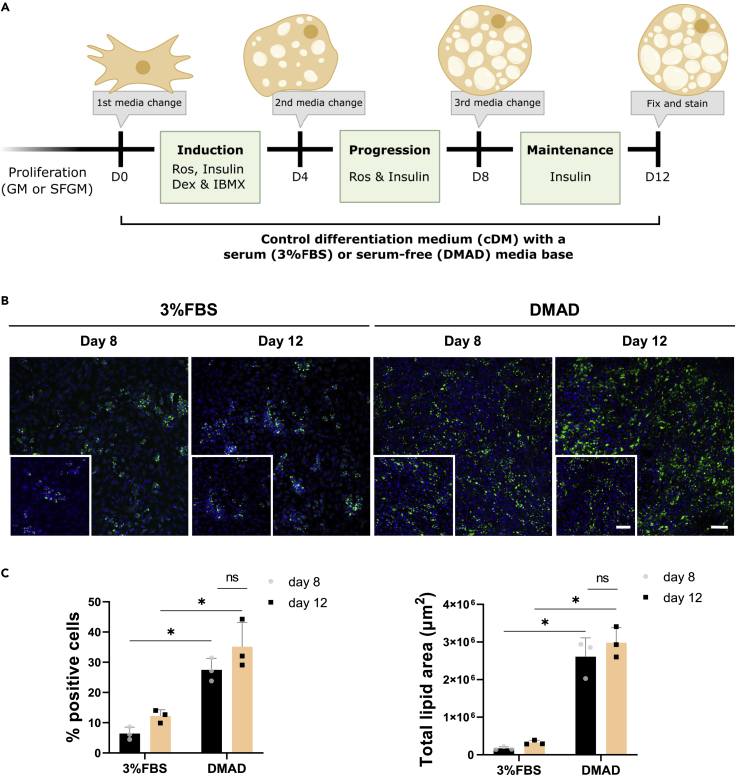
Differentiation in serum-free versus FBS-medium (A) Simplified schematic illustration of 2D adipocyte differentiation process. Bovine SVC cells were proliferated in either serum-containing growth medium (10% FBS; GM) or serum-free growth medium (SFGM). Adipogenesis was induced in a differentiation medium with 3%FBS or a defined animal component-free medium (DMAD). Media was changed 3 times. To assess the formation of adipocytes in response to the adipogenic cocktail in either serum or serum-free conditions, we performed fluorescent staining of the lipid droplets and nuclei, and quantified them with a high content analyzer ImageXpress Pico (HCA). Ros, rosiglitazone; and Dex, dexamethasone. (B) Representative fluorescent images of cells on day 8 of differentiation with 3%FBS or DMAD, stained with BODIPY (green lipid droplets) and Hoechst (blue nuclei). Scale bar, 100 μm. (C) Quantification of adipogenesis at day 8 and 12 with an HCA. Data are represented as mean ± standard deviation (SD); error bars represent the SD of *3* independent experiments using 3 donors. Statistical significance was determined with a two-way ANOVA; NS, not significant; ∗p < 0.05.

The main strategies used for *in vitro* adipocyte differentiation are the supplementation of free fatty acids (FA) and the so-called traditional adipogenic differentiation protocol or cocktail.^19^^,^^20^^,^^21^ The first one of which requires constant supply of a specific combination of free FA and can therefore be costly given the lengthy maturation period of adipocytes. An empirically derived adipogenic differentiation protocol is more commonly used and activates a signaling cascade and subsequent gene expression toward an adipocyte phenotype.^22^ It consists of a combination of three or four adipogenic inducers that are added to the medium typically in three phases: induction, progression and maintenance (Figure 1A). Induction (I) lasts from 2–4 days and signals to expanding cells to stop proliferating and start activating transcription factors which regulate adipogenesis.^23^ Induction medium has insulin, IBMX, dexamethasone and, depending on the investigators, rosiglitazone. Progression medium (P) lasts 2–4 days and usually has a combination of insulin and either rosiglitazone or dexamethasone. During maintenance (M, lasts 2–24 days) either insulin alone or no inducers are added. All pathways activated by adipogenic inducers lead to peroxisome proliferator-activated receptor gamma (PPARγ), through increasing its expression by the family of CCAAT enhancer binding proteins (C/EBP) with insulin, dexamethasone and IBMX; or its activity by ligands such as rosiglitazone.^24^

Adipogenesis is mostly studied in cell lines or primary cells from mice or human donors, and often in media containing fetal calf serum (FBS).^25^^,^^26^ These monogastric species have a lipid metabolism that is different from ruminants such as cows. For instance, in ruminants, lipogenesis mainly occurs in the fat tissue and the carbohydrate source for FA is acetate derived from enteric fermentation.^27^ Whereas in monogastric species, glucose derived acetate is the main carbon source, and FA biosynthesis occurs primarily in the liver.^28^ Differentiation of bovine adipogenic precursors using the standard adipogenic cocktail is less efficacious than in human or murine models and there is no consensus on the optimal protocol for bovine adipogenesis (Table S1). In addition, very little information on bovine adipogenesis in serum-free medium exists, which is a prerequisite for cultivated meat purposes.

Cultivated fat used for future consumption in a cultivated meat context will need to be produced with methods that are food safe. Although insulin is an endogenous protein, three of the differentiation inducers are synthetic: dexamethasone, 3-Isobutyl-1-methylxanthine (IBMX) and rosiglitazone. They were initially developed for treatment of cardiovascular/neurological, inflammatory diseases and diabetes.^29^^,^^30^^,^^31^ Of these, IBMX and rosiglitazone are toxic and therefore not food-compatible.^32^^,^^33^

In this study, we revisited the adipogenic protocol for bovine stromal vascular cells (SVCs) in the setting of a customized, serum-free, defined medium for adipogenic differentiation (DMAD). With a full-factorial experimental design, we investigated the necessity of adipogenic phases and compounds as well as their optimal concentrations. In addition, we questioned the relevance of distinguishing phases in adipogenesis and established a one-step differentiation protocol containing only 2 of the traditional inducers. We also explored the capacity of selected inducers to sustain adipocyte maturation in 3D long-term culture, their effect on FA profile and gene expression. To ascertain general applicability to other species we applied the changes to primary porcine and ovine adipogenic precursor cells, as well as murine (3T3-L1) cells.

## Results

### Serum-free differentiation outperforms serum

To investigate differentiation with the standard adipogenic cocktail, we firstly proliferated bovine SVCs in either serum-containing growth medium (10% FBS; GM) or serum-free growth medium (SFGM). Subsequently, adipogenesis was induced by a differentiation medium that either contained serum (hereinafter referred to as 3%FBS), if proliferated in serum, or DMAD, our in-house developed serum-free differentiation medium, if proliferated in SFGM. An FBS concentration of 3% was chosen for the serum containing differentiation medium as opposed to the commonly used 10%,^21^ as we found this to be the optimal concentration for bovine adipogenesis (data not shown). These results are in agreement with previous studies, which have shown that FBS may dose-dependently inhibit adipogenesis.^34^

It was observed that DMAD markedly outperforms the 3%FBS condition, as seen on both day 8 and 12 (Figure 1B), where both the percentage of positive cells and total lipid area were significantly higher in the serum-free condition (Figure 1C). Optimization of the adipogenic cocktail for bovine SVCs in these studies was thus performed using DMAD rather than serum-containing medium. After 12 days of culture, adipogenesis was not significantly increased when compared to day 8 (Figure 1C). Given that the additional 4 days of culture did not have a significant effect on differentiation quality, screening of adipogenic inducers during the maintenance phase/third media change was excluded. Omitting this step also allowed us to focus on developing a simplified adipogenic protocol.

### Rosiglitazone and insulin sufficiently induce adipogenesis

**Figure 2:**
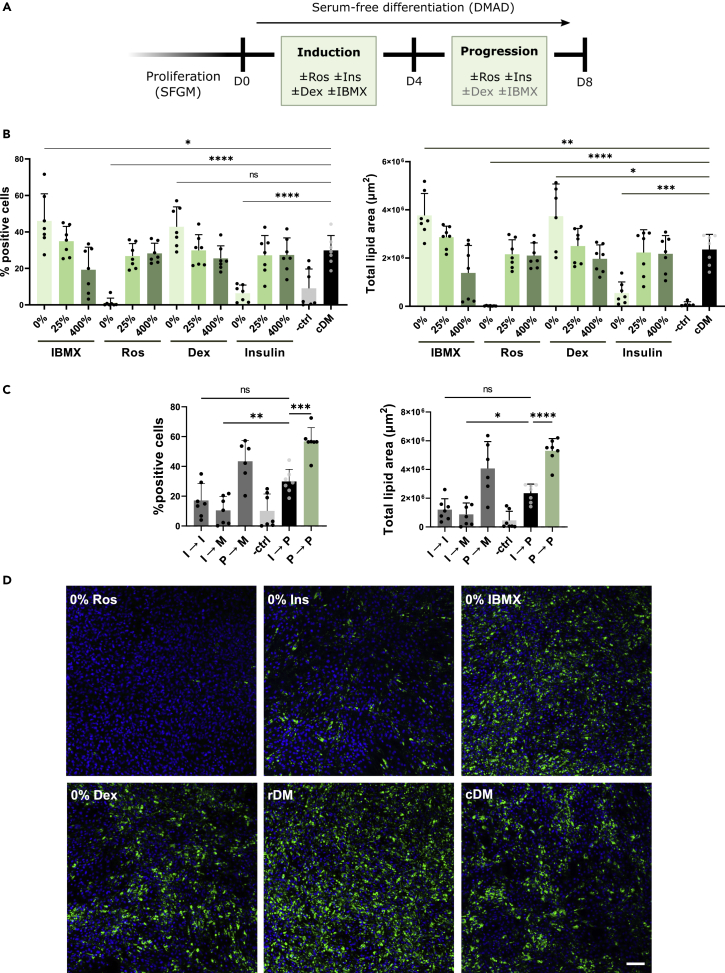
Rosiglitazone and insulin are the essential components of the differentiation cocktail (A) Illustration of the experimental design for 2b. Bovine SVC cells were proliferated in serum-free growth medium (SFGM) and differentiated with a defined animal component-free medium (DMAD). Varying concentrations or removal of adipogenic inducers: IBMX/dexamethasone (Dex) were tested during the induction phase; rosiglitazone (Ros)/insulin (Ins) were tested during the induction and progression phase. (B and C) Quantification of lipid accumulation with HCA shown at day 8. (B) Concentration gradients of differentiation media components were tested at 0, 25 and 400% of their standard concentrations. (C) Varying sequences of induction (I), progression (P) and maintenance (M) media. -ctrl, no inducers; I→P or control differentiation medium (cDM) has all 4 inducers in the first media change followed by Ros and Ins (P); *P*→P or reduced differentiation medium (rDM) contains only Ros and Ins as inducers. Data are represented as mean ± SD; the error bars represent the SD of *4* independent experiments using 4 donors. Statistical analyses and comparisons were performed using a one-way ANOVA; NS, not significant; ∗p < 0.05; ∗∗p < 0.01; ∗∗∗p < 0.001; and ∗∗∗∗p < 0.0001. (D) Representative images taken on day 8. Blue, Hoechst; and green, BODIPY. Scale bar, 100 μm. See also Figure S1.

Complete exclusion and three different concentrations of each component (rosiglitazone, insulin, IBMX and dexamethasone) were investigated, as visualized in Figure 2A. Removal of insulin or rosiglitazone from the traditional protocol (control differentiation medium; cDM) resulted in significantly decreased differentiation compared to the control, revealing their necessity (Figures 2B and 2D). In contrast, removing IBMX increased both the percentage of positive cells and total lipid area, whereas the removal of dexamethasone significantly increased lipid area but did not affect percentage of positive cells.

We also investigated the optimal sequence of the differentiation phases. As such, four key combinations were explored: (1) Induction followed by induction; (2) induction followed by maintenance; (3) progression followed by maintenance; and (4) progression followed by progression. It was observed that 8 days of only induction did not significantly affect differentiation; however, induction followed by maintenance reduced differentiation significantly (Figure 2C). Whereas progression followed by maintenance increased differentiation, and progression media for the entirety of culture (hereinafter referred to as reduced Differentiation Medium; rDM) resulted in the highest level of lipid accumulation (Figures 2C and 2D). This gives clear support that only the combination of rosiglitazone and insulin is required to induce differentiation, and that the inclusion of IBMX and dexamethasone is not needed, further implying that the distinction between induction and progression phase is no longer relevant. In addition, it re-emphasises that the maintenance medium does not increase adipogenic potential following induction or progression media.

### Hydrocortisone/progesterone are necessary for homogeneous cell distribution and health during differentiation

**Figure 3:**
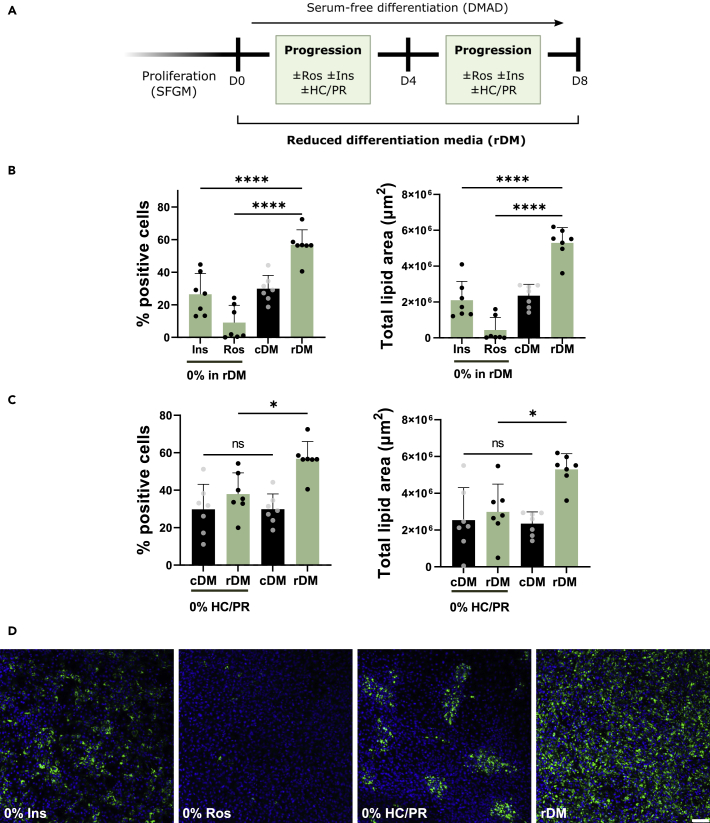
Rosiglitazone/insulin and hydrocortisone/progesterone are necessary for high quality differentiation (A) Illustration of the experimental design. Bovine SVC were proliferated in SFGM and differentiated with DMAD. Rosiglitazone and insulin were removed from the reduced differentiation media (rDM). Hydrocortisone and progesterone (HC/PR) were also excluded because they are reported to activate GCR, same as dexamethasone. (B) Screening the necessity of rosiglitazone (Ros) and insulin (Ins) within rDM. Adipogenic quantification at day 8. (C) HC/PR were excluded from rDM or control differentiation media (cDM). In cDM-HC/PR, IBMX was present during the first 4 days, and dexamethasone was absent entirely. (B and C) Data are represented as mean ± SD; the error bars represent the SD of *4* independent experiments using 4 isolations. Statistical analyses were performed using a one-way ANOVA; ∗p < 0.05; ∗∗∗p < 0.001; and ∗∗∗∗p < 0.0001. (D) Representative images taken on day 8. Blue, Hoechst; and green, BODIPY. Scale bar, 100 μm. See also Figure S2.

The combination of insulin and rosiglitazone is sufficient for adipogenesis as seen in Figure 2, however rosiglitazone or insulin as inducers alone were not tested. As such rDM was tested when either insulin (rDM, 0% Ins) or rosiglitazone (rDM, 0% Ros) was removed for the 8 days of culture (see illustration of experimental design in Figure 3A). The absence of rosiglitazone almost completely diminished any differentiation, whereas no insulin more than halved it (Figures 3B and 3C). Both inducers are therefore necessary components of rDM.

As dexamethasone, a glucocorticoid receptor (GCR) activator, appeared to negatively affect adipogenesis, we further investigated the presence of hydrocortisone and progesterone (HC/PR), two endogenously present GCR activators in DMAD. We observed that removing all GCR binding molecules in ‘rDM, 0% HC/PR’ (removal of hydrocortisone and progesterone) resulted in significantly reduced differentiation (Figure 3C). However, ‘cDM, 0% HC/PR’ (removal of dexamethasone, hydrocortisone, and progesterone but with IBMX), did not have significantly lower adipogenesis compared to cDM. In both cases however, the removal of hydrocortisone and progesterone lead to cells that differentiated in clusters and presented unhealthy morphology or even died in certain isolations (Figures 3D and S2). This suggests that GCR binding molecules, at relatively low concentrations, are important components of adipogenic differentiation media for homogeneous differentiation, as well as for cell viability and spreading.

### Reduced differentiation medium induces a high level of adipogenic markers

**Figure 4:**
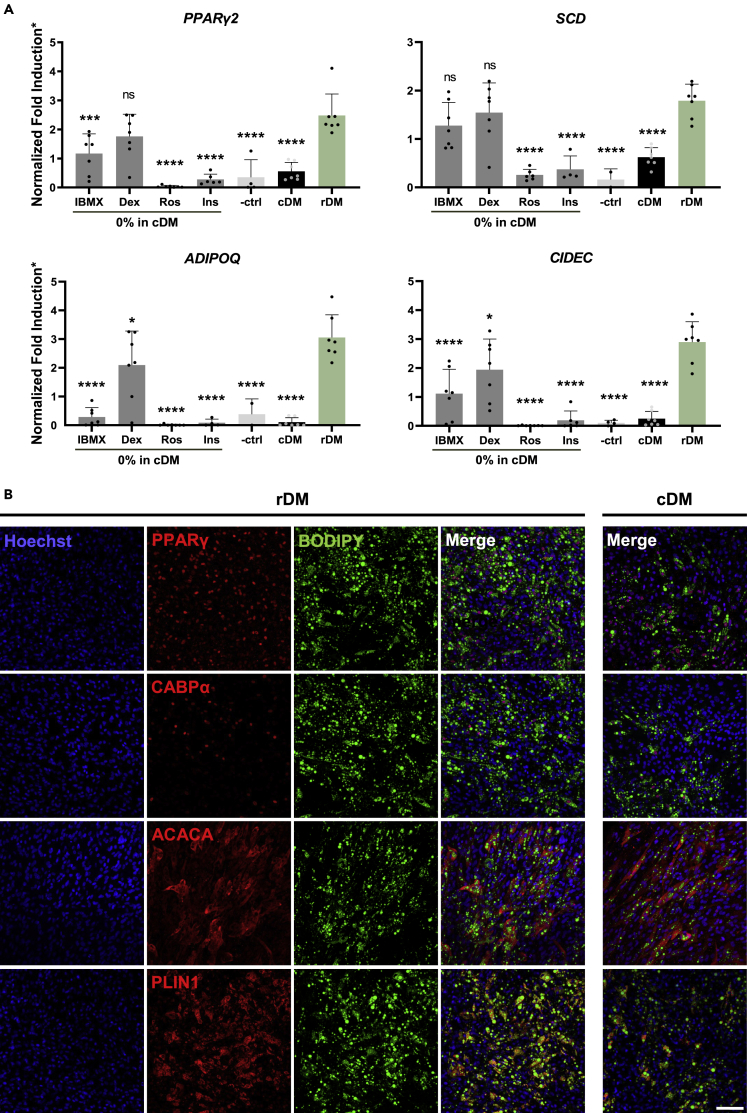
Cells differentiated with rDM present high expression of adipogenic markers at the gene and protein level (A) Mean gene expression fold changes 12 days after induction of bovine SVC differentiation, determined by qPCR. Adiponectin (*ADIPOQ*), cell death activator (*CIDEC*), and stearoyl-CoA desaturase (*SCD*). All conditions were normalized to a chosen set of reference genes (*UXT, RPLP, L19*) and to day 0 control (not shown); ∗mean 2^-^^ΔΔ^^Ct^ values of each condition were divided by the average of same isolation to improve comparability between conditions. Data are represented as mean ± SD; the error bars represent the SD of 4 independent experiments using 4 donors. One-way ANOVA; NS, not significant; ∗p < 0.05; ∗∗∗p < 0.001; and ∗∗∗∗p < 0.0001. All conditions are compared to rDM. (B) Immunohistochemical analysis of bovine SVC differentiated in rDM for 8 days. PPARγ and CEBPA are early differentiation markers; Acetyl-CoA carboxylase 1 (ACACA) and Perilipin 1 (PLIN1) are maturation markers. Scale bar, 100 μm. See also Figures S3 and S4.

To verify that rDM results in adipogenic marker expression, we investigated their presence at the gene and protein level. For the gene expression panel, early differentiation- *PPAR*γ*2*; and adipocyte maturation markers- *ADIPOQ, CIDEC* and *SCD* were analyzed. For statistical analysis all conditions were compared to rDM. qPCR results confirmed significantly greater upregulation of all adipogenic genes except SCD in cells differentiated with DMAD rDM than with cDM (Figure 4A). Differentiation in 3%FBS did not demonstrate significant difference in gene expression between rDM and cDM in case of *SCD* and *ADIPOQ* and was better in case of *PPARγ* and *CIDEC*, suggesting that rDM can be used in combination with different media (Figure S3). Removal of IBMX from cDM did not result in a significant difference of *SCD* when compared to rDM, whereas *PPAR*γ*, CIDEC* and *ADIPOQ* were downregulated. Similarly, taking out dexamethasone from cDM did not significantly decrease the expression of *SCD* and *PPAR*γ when compared to rDM, however *CIDEC* and *ADIPOQ* were significantly downregulated. Removing insulin and rosiglitazone on the other hand resulted in significantly lowered expression of all four genes. This confirms our initial observation that dexamethasone and IBMX are redundant in our adipogenic differentiation media.

Furthermore, to confirm adipocyte differentiation with rDM at a protein level, we investigated the presence of PPARy and CEBPα (early differentiation markers), and ACACA and PLIN1 (maturation markers). Consistent with the qPCR data, adipogenic proteins are clearly expressed (Figure 4B). Qualitatively, cDM appears to result in lower amounts of adipogenic proteins than rDM overall (Figures 4B and S4). Together, this data indicates that cells can differentiate into mature adipocytes with rDM, warranting it as an adipogenic cocktail for cultivated fat.

### Reduced differentiation medium supports the cultivation of 3D adipogenic microfibers

**Figure 5:**
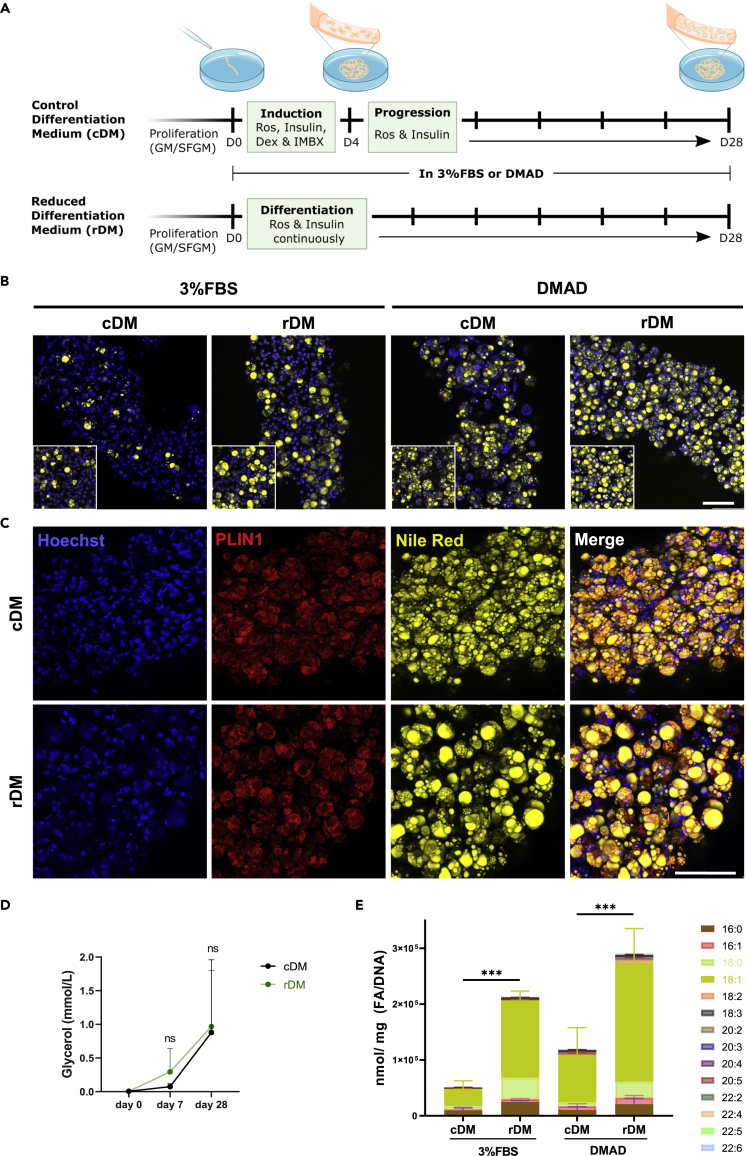
rDM outperforms cDM in both 3%FBS and DMAD during long-term (28-day) 3D culture (A) Illustration of the experimental design. Bovine SVC were encapsulated with alginate hydrogel and differentiated with 3%FBS or DMAD. cDM contained all 4 inducers during first media change followed by progression for the whole period of culture, rDM contained rosiglitazone and insulin for 28 days. DMAD contained HC/PR. (B) Representative images of maximum intensity projection confocal microscopy. Differentiation with control (cDM) or reduced (rDM) differentiation medium in 3%FBS or DMAD at day 28. Blue, Hoechst; and yellow, Nile Red. (C) PLIN1 immunohistochemistry of 3D hydrogel/cell constructs differentiated in cDM or rDM medium at day 28. Blue, Hoechst; red, PLIN1; and yellow, Nile red. (B and C) Scale bar, 100 μm. (D) Quantitative analysis of glycerol release at day 0, 7 and 28. Data is representative of 3 independent experiments using 4 donors. (E) Qualitative and quantitative assessment of the amount of fatty acids within triglycerides (normalized to the amount of DNA). Results are representative of 5 independent experiments using 6 donors. (D and E) Statistical differences were analyzed with a one-way ANOVA. Data are represented as mean ± SD; NS, not significant; ∗∗∗p < 0.001. See also Figure S5 and S6.

We compared the newly developed one-step rDM protocol to cDM in 3D adipogenic microfibers. 3D cultures were studied as they are a representative model of large-scale cultivated fat production. The 3D constructs were differentiated for 4 weeks with either rDM or the traditional inducers (cDM) in serum (3%FBS) or serum-free (DMAD) conditions, as illustrated in Figure 5A. Differentiation quality was assessed via confocal microscopy, glycerol release and lipidomic profiling.

At week 1, 2D adipocyte cultures appear to have more lipid droplets than 3D alginate microfibers (Figure S5A), likely because of a lack of interaction between cells in the non-functionalized alginate. However, given that 2D adipocyte cultures have the tendency to detach in long-term cultures (∼28 days), 3D constructs are required.

Assessment at week 1 of the microfiber culture visibly shows that the cells start to differentiate with higher efficiency with rDM when compared to cDM (Figure S5A). This trend continues onto week 4, where rDM results in an increased number of differentiating cells and bigger lipid droplet diameter than cDM in both DMAD and 3%FBS (Figure 5B). Cells within the microfibers differentiated with both rDM and cDM showed a strong signal for PLIN1 (Figure 5C). Glycerol release, typically used to study triglyceride/FA cycling, revealed glycerol concentration increased with time of culture in both rDM and cDM, with no significant difference between the two conditions (Figure 5D).

With lipidomic profiling we confirmed the qualitative observations in Figure 5B: rDM significantly outperforms cDM in both serum and serum-free media in total lipid content (Figures 5E and S5C). Subsequently we compared the triglyceride composition of conventional and cultivated fat (Figure S6). Bovine fat had a significantly higher amount of palmitic (16:0) and linoleic acid (18:2) compared to all cultivated samples (Figure 5E). Depending on the *in vitro* condition, cultivated fat contained either a similar amount of stearic acid (18:0) (cDM DMAD), lower (rDM DMAD/FBS), or higher amounts (rDM FBS) compared to bovine fat. Oleic acid (18:1) is significantly higher in cultivated fat compared to bovine fat. Long chain polyunsaturated fatty acids (PUFAs) are absent in bovine fat but detectable in fat cultured with DMAD, in particular in the cDM condition.

### Transcriptomic investigation of the rDM inducers

**Figure 6:**
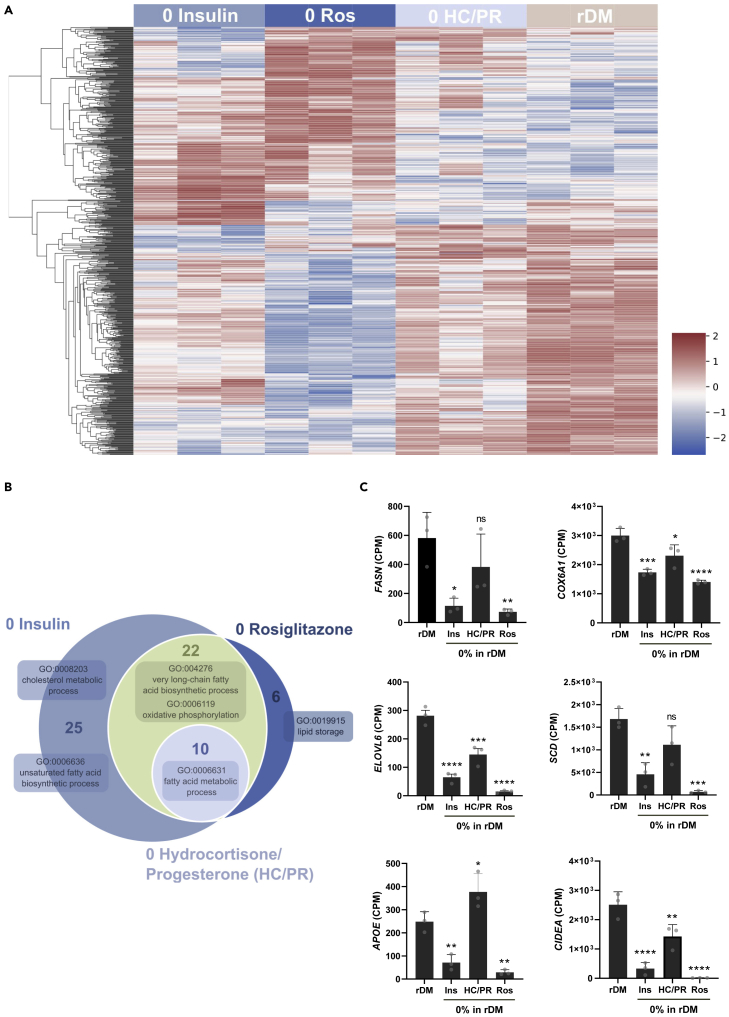
Transcriptomic analysis demonstrates the impact of insulin, rosiglitazone and glucocorticoid receptor binding molecules on differentiating adipocytes (A-C) Bovine SVC were proliferated in SFGM and differentiated with DMAD rDM, rDM 0% Ins, 0% Ros or 0% HC/PR. mRNA was harvested at day 12. 3 donors were used in 3 independent experiments. (A) Heatmap showing the *Z* score normalized expression values of all 894 genes that were differentially expressed in at least one comparison. (B) Venn diagram showing overlapping GO terms corresponding to upregulated genes in rDM when compared to rDM deprived from insulin, rosiglitazone, or HC/PR. (C) Selection of differentially expressed genes of indicated GO terms. Fatty acid synthase (*FASN*) is involved in FA synthesis (metabolic process); Cell death activator CIDE-A (*CIDEA*) involved in lipid droplet enlargement (lipid storage); *ELOVL6* plays a role in very long chain fatty acid elongation; Stearoyl-CoA desaturase (*SCD*) is involved in the generation of unsaturated FA; Apolipoprotein E (*APOE*) is involved in cholesterol metabolism and fat intratissue transportation; and Cytochrome *c* oxidase subunit 6A1 (*COX6A1*) is involved in oxidative phosphorylation. Data are represented as mean ± SD. Statistical analyses and comparisons were performed using a two-way ANOVA; NS, not significant; ∗p < 0.05; ∗∗p < 0.01; ∗∗∗p < 0.001; and ∗∗∗∗p < 0.0001. See also Figure S7.

**Figure 7:**
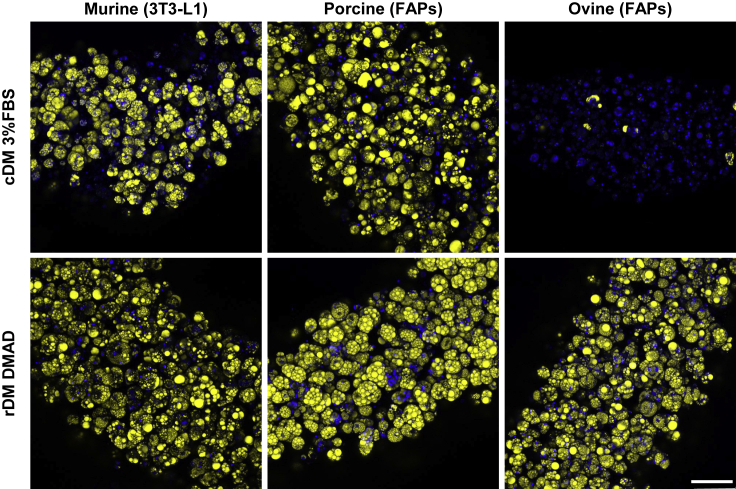
Adipogenic differentiation of different species: mouse 3T3-L1 cell line; porcine FAPs (monogastric); and sheep FAPs (ruminant) Differentiation with control (cDM in 3%FBS) or reduced (rDM in DMAD) differentiation medium at day 28. Cells were encapsulated with alginate hydrogel and differentiated with 3%FBS or DMAD. 3%FBS cDM contained all 4 inducers during first media change followed by progression for the whole period of culture, rDM contained rosiglitazone and insulin for 28 days. DMAD contained HC/PR. n = 3 donors. Representative images of maximum intensity projection confocal microscopy (Blue, Hoechst; and yellow, Nile Red). Scale bar, 100 μm.

To characterize and compare the changes at the gene expression level that each essential inducer has on adipogenesis, we have differentiated bovine SVC with rDM or rDM deprived from insulin, rosiglitazone, and HC/PR, and performed RNAseq on these samples. We observed that taking out rosiglitazone and insulin resulted in large changes in gene expression levels when compared to rDM, whereas taking out HC/PR showed a more comparable pattern to rDM and less changes at the gene expression level (Figure 6A). This is in line with the phenotypic effect on differentiation that insulin and rosiglitazone exhibit on bovine SVC. Enriched gene ontology (GO) terms indicated the crucial role of insulin and rosiglitazone in upregulating the expression of genes related to adipocyte phenotype and highlighted their overlapping effect on gene expression (Figure 6B). rDM compared to rDM without insulin results in 57 significantly enriched upregulated GO terms related mainly to FA and lipid metabolism, oxidative phosphorylation, and cholesterol metabolism (Figure S7). Rosiglitazone results in 38 GO terms mainly involved in lipid metabolism and HC/PR in 10 enriched GO terms related to aerobic respiration and FA metabolic processes. From the list of shared GO terms, we focused on the most prominently present terms indicated in Figure 7B. With multiple markers we observed that rDM shows upregulation of FA storing or metabolism related genes (*CIDEA*, *SCD*, *ELOVL6*, *FASN*) as well as FA and cholesterol transportation (*APOE*) and numerous genes involved in aerobic respiration (*COX6A1*). Genes which are reported to be involved in the transmission of the signal generated by adipogenic inducers (*FOXO1*, *CREB1*, *NR3C1*) did not show significant upregulation (Figure S7). In addition, we observed that cells differentiated with rDM do not show expression of the brown fat marker *UCP-1*, indicating that our media selectively induces white adipogenesis over brown.

### rDM robustly supports differentiation across various species

Studies on monogastric species (mouse and human) have been crucial for understanding adipogenesis and developing traditionally used protocols (cDM in medium containing serum). However, these protocols do not allow for efficient *in vitro* differentiation of ruminant species such as cows, possibly due to significant differences between monogastric and ruminant physiology and lipid metabolism. Contrarily rDM performs well in differentiating bovine SVCs, thus for understanding whether reduced inducers are species specific and if they could be used for application in other cultivated meat products, we have assessed the translation of our one-step protocol to another ruminant animal (sheep) and two monogastric species (pig and mouse). For this we compared the capacity of rDM to efficiently differentiate ovine and porcine FAP cells, as well as murine 3T3-L1 cells. Cells derived from the monogastric species, pig and mouse, differentiated efficiently in cDM 3%FBS (Figure 7), whereas sheep cells did not, much like cow cells (Figure 5). Although cells from all three species, conversely, underwent high levels of adipogenesis with rDM DMAD. According to these results, rDM is not species specific, as opposed to the traditional protocol using FBS and the four conventional inducers.

## Discussion

For initiating adipogenesis in cell culture, a cocktail of inducers containing insulin, IBMX and dexamethasone, and frequently rosiglitazone has been reported as necessary. Predominantly these studies used mouse or human cell lines in serum-containing media. Here, we report superior adipogenesis in bovine SVCs in serum-free, chemically defined, differentiation medium in 2D and 3D. In this medium, which contains a relatively low amount of steroids, dexamethasone has no additional adipogenic activity. IBMX, a substance that is toxic for humans and therefore not food-compatible, could also be eliminated from the adipogenic cocktail, leaving only insulin and rosiglitazone as the required adipogenic inducers.

Understanding of adipogenic differentiation started with work on mouse embryonic fibroblasts and development of the self-differentiating 3T3-L1 cell line.^35^^,^^36^ With addition of insulin, IBMX and dexamethasone, differentiation time shortened from 4 weeks to 6–7 days, and adipogenesis increased presenting up to 90% positive cells.^37^ Phenotype reproducibility and lack of donor-to-donor variability of 3T3-L1 was of great importance in shaping and accelerating the knowledge behind adipocyte differentiation. However, the protocol has been adopted across different species and cell types with varying efficacy. For example, mouse embryonic cell lines including 3T3-442A, 1246, Ob1771 require different inducers for optimal differentiation^26^^,^^38^^,^^39^ and the differentiation of ruminant adipogenic precursors using the traditional protocol shows low efficacy.

Most adipogenic differentiation studies were performed using FBS. Besides batch-to-batch variability, ethical concerns and unknown composition, serum contains molecules involved in adipogenic differentiation (insulin ≈10 μU/mL, cortisone ≈ up to 54 ng/mL, progesterone ≈ up to 80 ng/mL), leading to difficulties in studying the inducers.^40^^,^^41^^,^^42^ Although several investigators have explored inducer necessity in serum,^21^^,^^43^^,^^44^ the few reports on efficient differentiation in a serum-free environment^45^^,^^46^^,^^47^^,^^48^^,^^49^ were limited to the standard adipogenic protocol. Using full factorial design in serum-free medium (Table S3) we showed that IBMX and dexamethasone can be excluded from the differentiation media.

IBMX is a phosphodiesterase inhibitor which increases intracellular cAMP, known to play a key role in 3T3-L1 differentiation.^50^ In our hands, supplementation of IBMX during induction did not benefit differentiation; on the contrary, increasing its concentration or exposure time resulted in cell detachment and death, in line with previous studies.^50^^,^^51^ IBMX was long reported to act on adipogenesis through the increase of C/EBPβ expression via cAMP response element-binding protein (CREB).^52^^,^^53^ Later, it was discovered that cAMP-induced signaling overlaps with insulin-induced signaling^54^^,^^55^ supporting our observation that IBMX does not have an additive effect as an inducer.

Elevated local levels of glucocorticoids in fat tissue are reported to result in increased obesity and metabolic syndrome,^56^ however these observations are conflicting between species or tissue depots.^57^^,^^58^ Dexamethasone is a synthetic glucocorticoid which binds to the GCR and acts on CEBPδ and CEBPβ.^59^^,^^60^^,^^61^ Its endogenous alternatives hydrocortisone and progesterone,^62^ which are present in FBS, are reported to have lower activity on GCR^37^ and differentiation potential compared to dexamethasone; dexamethasone is therefore still supplemented across adipogenic protocols. Of interest, we observed that leaving out dexamethasone did not have an impact on differentiation quality. However, because DMAD contains hydrocortisone and progesterone (at concentrations comparable to 10% FBS and slightly higher than 3% FBS - up to 4.47 nM HC and 7.6 nM PR in 3% FBS, versus 25 nM HC and 17 nM PR in DMAD^40^^,^^42^), omitting only dexamethasone hindered the assessment of GC signaling during adipogenesis. After depleting DMAD from all the GCR activators, we observed lower differentiation, with cells presenting unhealthy morphology, lower cell numbers and differentiation in islands. However, excluding or including hydrocortisone and progesterone from rDM did not result in a large change at the adipogenic gene expression level. The importance of hydrocortisone in cell attachment, spreading and proliferation of SVC has been documented in several serum-free environments,^63^^,^^64^ our findings similarly imply that GCR-induced signaling might be more important for cell survival related mechanisms than adipogenesis.^61^^,^^65^^,^^66^ The inclusion of dexamethasone and IBMX therefore does not appear necessary for adipocyte differentiation.

Insulin was the first adipogenic stimulator discovered and in the absence of other inducers it is reported to ameliorate differentiation of 3T3-L1 cells in a concentration dependent manner.^67^ In our culture, removing insulin from cDM or rDM resulted in few differentiating cells containing small lipid droplets. The presence of insulin showed significant upregulation of a multitude of adipogenic and lipogenic genes, it also indicated its importance in cholesterol metabolism and oxidative phosphorylation during differentiation. A great overlap of enriched upregulated GO terms when insulin or a PPARγ agonist are supplemented points out the converging mechanisms that these two have in promoting adipocyte differentiation. Our observations are in line with numerous previous studies in different species where insulin importance was demonstrated *in vitro* and *in vivo.*^68^^,^^69^

The beneficial effects of PPARγ activation on adipogenesis were observed when the PPARγ agonist, indomethacin, induced high levels of differentiation in 3T3-L1 cells.^37^^,^^70^ Subsequently, highly specific PPARγ agonists were developed for the treatment of diabetes,^71^ and notably rosiglitazone emerged as a potent adipogenic stimulator.^72^ Similarly, we observed that rosiglitazone effectively induced differentiation, whereas its absence resulted in minimal adipogenesis. Unlike other factors, in 2D culture it is sufficient to induce adipogenesis in a relatively small number of cells in the absence of all other inducers. However, in our 3D system, DMAD negative control showed a discrete number of cells containing large lipid droplets (Figure S5B). This is contrary to our observations in the 2D short-term culture, where the negative control (containing insulin and HC/PR, but no PPARγ agonist) did not differentiate. Differentiation is possibly driven by our media composition and the presence of lipid concentrate in DMAD, where fatty acids such as linoleic and oleic are known to have a weak PPARγ activating function.^19^^,^^20^ Stronger PPARγ agonists, such as rosiglitazone, are likely necessary to increase the percentage of differentiating cells. The pro-adipogenic effect of rosiglitazone was supported at the gene expression level where the highest number of differentially expressed genes was present when rDM was deprived from rosiglitazone. Therefore, a PPARγ agonist is necessary to induce adipogenesis of bovine SVCs.

For an appealing cultivated meat product, besides a low-cost, animal component-free, safe media, cultivated fat should mimic the nutritional and sensory qualities of conventional beef fat. For this purpose, we performed lipidomic investigation of cultivated fat and cow derived fat. We focused on the FA composition within triglycerides because FA species have a major effect on the sensory and nutritional value of fat.^15^ Stearic acid has been reported to greatly impact the firmness and the melting point of bovine fat.^14^^,^^73^ Cultivated fat differentiated with rDM DMAD did not show significant differences in stearic acid compared to conventional bovine fat. Oleic acid has been reported to greatly impact taste and high amounts improve sensory quality.^74^ Of interest, *in vitro* grown adipocytes from all four media have much higher amounts of oleic acid compared to bovine fat, an observation with unclear underlying mechanism. PUFAs have a major impact on human health. It has been well documented across the literature that bovine fat does not contain long chain PUFA within neutral lipids.^14^ In cells grown with DMAD several species of PUFA were present. rDM did not show significantly higher amounts of EPA and DHA however cDM had higher amounts of all the members of long chain PUFA when compared to bovine fat. This exciting result suggests that *in vitro* grown adipocytes, if cultured with food compatible signaling molecules, can result in healthier fat for human consumption.

In addition, to the best of our knowledge, our reduced defined medium is the first to demonstrate high differentiation level across four species and is comparable (monogastric) or better (ruminant) than the traditional cocktail in FBS containing medium. Having a medium able to differentiate adipogenic precursors originating from different tissues^75^ and domestic animals is of great importance for making cultivated fat. We have yet to determine how DMAD is able to overcome physiological differences reported during adipogenesis between species, *in vivo* and in FBS.

With our study we established a new, simplified one-step adipogenic protocol with a reduced number of adipogenic inducers and phases. The most important inducers are rosiglitazone and insulin, whereas GCR activators ameliorate differentiation quality. Our newly developed protocol in an animal component-free defined medium with the reduced number of inducers can be used across two monogastric and two ruminant species for high quality differentiation. This work has narrowed the search for finding food safe inducers to only a PPARγ agonist before establishing a compatible media for cultivated fat production.

### Limitations of the study

This study investigated changes at the gene expression level that each inducer has on adipogenesis. To add to the understanding of signaling pathways activated by each inducer within the new rDM medium, future work should focus also on the changes occurring at a protein level. In addition, we demonstrate that although the rDM induces and sustains long-term 3D differentiation, the timings and necessity of each rDM inducer during the entire 28-day culture were not investigated. Lastly, rosiglitazone is not a food compatible component and thus cannot be used for cultured fat production, requiring an alternative. These questions would form the basis of future studies into rDM.

## STAR★Methods

### Key resources table

**Table:** 

REAGENT or RESOURCE	SOURCE	IDENTIFIER
**Antibodies**		

anti-rabbit Alexa 594	Thermo Fisher	Donkey rAb Cat #R37119; RRID:AB_2556547
anti-mouse ACACA	Cell signaling technology	Rabbit mAb Cat #3676; RRID:AB_2219397
anti-mouse CEBPɑ	Cell signalling technology	Rabbit mAb Cat #8178; RRID:AB_11178517
anti-mouse PLIN1	Cell signalling technology	Rabbit mAb Cat #9349; RRID:AB_10829911
anti-mouse PPARγ	Cell signalling technology	Rabbit mAb Cat #2435; RRID:AB_2166051

**Biological samples**		

Bovine subcutaneous adipose tissue	Local abattoir	N/A
Porcine semitendinosus muscle	Local abattoir	N/A
Ovine semitendinosus muscle	Local abattoir	N/A

**Chemicals, peptides and recombinant proteins**		

DMEM/F12	Gibco	Cat# 21331-020
DMEM	Gibco	Cat# 41966-029
PSA	Lonza	Cat# 17-745E
FBS	Gibco	Cat# 10500-064
Trypsin-EDTA	Gibco	Cat# 25300-062
Glutamine	Gibco	Cat# 35050-061
Lipid concentrate	Gibco	Cat# 11905-031
HEPES	Sigma-Aldrich	Cat# H4034
Putrescine	Sigma-Aldrich	Cat# 51799
Progesterone	Sigma-Aldrich	Cat# P8783
Hydrocortisone	Sigma-Aldrich	Cat# H0135
Ascorbic acid	Sigma-Aldrich	Cat# A8960
BMP4	Peprotech	Cat# 120-05ET
FGF2	Peprotech	Cat# 100-18B
EGF	Peprotech	Cat# AF-100-15
Insulin	Sigma-Aldrich	Cat# I0516
Rosiglitazone	Sigma-Aldrich	Cat# R2408
Dexamethasone	Sigma-Aldrich	Cat# D1756
IBMX	Sigma-Aldrich	Cat# I5879
Alginate	Sigma-Aldrich	Cat# W201502
Bodipy	Thermo Fisher	Cat# D3922
Hoechst 34,580	Sigma-Aldrich	Cat# 63493
Nile-red	Sigma-Aldrich	Cat# N3013
Collagenase	Worthington	Cat# CLSAFA
CaCl2	Sigma-Aldrich	Cat# C3881
iQ SYBR Green Supermix	Bio-Rad	Cat# 1708880
PBS	Gibco	Cat# 10010023

**Critical commercial assays**		

E.Z.N.A Total RNA kit II	Omega Bio-tek	Cat# R6934
iScript cDNA synthesis kit	Bio-Rad	Cat# 1708891
Dynabeads mRNA DIRECT™ MicroKit	Thermo Fisher	Cat# 61021
PCR-cDNA Barcoding kit	Oxford Nanopore Technologies	Cat# SQK-PCB109

**Deposited data**		

RNA sequencing	This study	GEO: GSE206990

**Experimental models: Cell lines**		

3T3-L1	Green et al., 1974	RRID:CVCL_0123

**Oligonucleotides**		

Table S2	Eurogentec	N/A

**Software and algorithms**		

GraphPad Prism 9.1.0	GraphPad Software	https://www.graphpad.com/scientific-software/prism/
Inkscape	Inkscape software	https://inkscape.org/
Guppy 5.0.16	Oxford Nanopore Technologies	nanoporetech.com
Minimap2	https://github.com/lh3/minimap2	N/A
Salmon	https://github.com/COMBINE-lab/salmon	N/A
DEseq2	https://bioconductor.org/packages/release/bioc/html/DESeq2.html	N/A
tximport	https://bioconductor.org/packages/release/bioc/html/tximport.html	N/A
SVA	https://bioconductor.org/packages/release/bioc/html/sva.html	N/A
Piano	https://bioconductor.org/packages/release/bioc/html/piano.html	N/A

### Resource availability

#### Lead contact

Further information and requests for resources and reagents should be directed to and will be fulfilled by the lead contact, Dr. Laura Jackisch (laura@mosameat.com).

#### Materials availability

This study did not generate new unique reagents.

### Experimental model and subject details

#### Primary bovine SVCs and ovine and porcine FAPs

Bovine/porcine/ovine primary cells were used in this study. Subcutaneous fat (for isolating SVCs) or semitendinosus muscle samples (for isolating FAPs) were obtained from a registered abattoir according to national guidelines on handling of animal by-products. Cells were isolated as described in the method details. In case of both bovine and porcine cells, female donors were used. Bovine donors were from 2 to 6 yearsold upon sacrificing and porcine up to one year. Sheep cells are from unknown donor sex and age because of the entry restrictions within this specific slaughterhouse.

#### Cell line

Mouse 3T3-L1 cell line was used for this study, RRID: CVCL_0123. Cells were treated in the same way as primary cells.

### Method details

#### Isolation of stromal vascular cells

Bovine subcutaneous fat samples were obtained from a registered abattoir according to national guidelines on handling of animal by-products. Ethical approval was not required for acquisition of tissue samples from commercially slaughtered cattle. Samples were acquired and transported in accordance with Dutch national regulations on handling animal by-products. Mosa Meat B.V. has a licence to handle Category 3 animal materials.

Stromal vascular cells (SVC) cells were isolated from the subcutaneous brisket fat of Belgian Blue cattle (both male and female, aged 1 to 7 years) as previously described.^19^^,^^75^ Briefly, fat tissue was minced and dissociated with collagenase (CLSAFA, Worthington; 2 h, 37°C). Cell pellets containing SVC and the supernatant containing mature adipocytes were washed with PBS, centrifuged again and pellets were filtered through a 100 μm cell strainer. Filtered cells were resuspended in growth medium (GM; see Table S3) or in defined, in-house developed, serum-free proliferation medium (SFGM; see Table S3) and seeded (10 grams of initial starting tissue per 75 cm^2^) in cell culture flasks for propagation.

#### Isolation of ovine and porcine adipogenic precursor cells

Fresh ovine and porcine skeletal muscle was obtained from a registered abattoir according to national guidelines on animal tissue handling. Muscle-derived adipogenic precursors (referred to as Fibro-adipogenic progenitor cells (FAP) from here on) were isolated as previously described.^76^ Semitendinosus muscle was minced and dissociated with collagenase (CLSAFA, Worthington; 1 h, 37°C). Cell slurries were filtered through a 100 μm cell strainer and incubated in ammonium-chloride-potassium (ACK) erythrocyte lysis buffer (1 min, room temperature (RT)). Cells were resuspended in GM, filtered through a 40 μm strainer prior to culture and seeded (10 grams of initial starting tissue per 75 cm^2^) in cell culture flasks. Murine, porcine, and ovine cells were grown in GM.

#### Cell culture

Cells were grown on tissue culture-coated culture dishes (Corning) in GM or in SFGM.^77^ For serial expansion, cells were cultured until 80–90% confluent, incubated with preheated trypsin-EDTA 1X (Gibco, 25300-062) at 37°C for 4 min. Trypsin was inactivated with GM/SFGM. Detached cells were then centrifuged at 350 × g for 5 min, resuspended in 1 mL and mounted on a counting chamber using trypan blue. Cell counting and viability estimation were performed with an automatic cell counter Countess (Thermo Fisher). For serial passaging, cells were seeded at a density of 5×10^3^ cells/cm^2^, incubated at 37°C, 5% CO_2_ and passaged at 80% of confluency.

#### Cell differentiation into adipocyte lineage

SVCs were differentiated at passage 2, 3 and 4, porcine (*Sus scrofa* *domesticus*) and ovine (*Ovis aries*) FAPs at passage 2, and 3T3-L1 cells at passage 7. For 2D differentiation cells were seeded at a density of 3×10^4^ cells/cm^2^ and proliferated for 1 day. Following this, either DMAD (Table S3) or 3%FBS differentiation medium (Table S3) were added to the cells, supplemented with different combinations/concentrations/timings of adipogenic inducers. In all cases of differentiation, cells proliferated in serum (GM) were differentiated in 3%FBS differentiation medium and cells proliferated in serum-free medium (SFGM) were differentiated in DMAD medium. Adipogenic inducers consist of insulin 10 μg/mL (Sigma; I0516); dexamethasone 1 μM (Sigma; D4902); IBMX 0.5 mM (Sigma; I5879) and rosiglitazone 5 μM (Sigma; R2408). First medium change containing all four inducers is referred to as induction medium, second medium change containing rosiglitazone and insulin as progression medium and third medium change without inducers as maintenance medium. Medium was changed every 4 days. The media containing all four components in the induction, insulin and rosiglitazone in progression, and maintenance with only insulin was our control (cDM; based on a protocol by Zhou et al., 2010^78^). To investigate inducer necessity, we did a full factorial design, that is we have tested all the possible inducer combinations (Figures 2 and S1) in the first two media changes (no software was used for the design). All 2D differentiation experiments were carried out in a Falcon 96-well plate with a well surface of 0.34 cm^2^. Details of the 3D differentiation cultures are given below.

#### Adipogenic microfiber cultures

SVCs were resuspended at a concentration of 6×10^7^ cells/mL in the respective differentiation medium. The cell suspension was mixed at a 1:1 v/v ratio with 1% alginate T1 H2O, to reach a final concentration of 0.5% alginate and 3×10^7^ cells/mL. Using a pipette, 100 μL of the cell-alginate suspension was injected into 66 mM CaCl_2_ in a 10 mM HEPES buffer to form cell-laden hydrogel microfibres with a diameter of 0.5 to 0.8 mm. The fibres were briefly washed in basal medium and transferred to a 12-well tissue culture plate containing 3 mL of DMAD or 3%FBS with (differentiation medium) or without (negative control; -ctrl) adipogenic inducers. After initial induction and/or progression medium, medium exchange was performed every 3–4 days supplementing progression medium for 28 days. Negative control did not contain inducers from the start of the culture. All fibres were incubated on a shaking platform (for the diffusion of gases and nutrients) at 75 RPM at 37°C with 5% CO_2_.

#### Lipid staining in 2D

Cells were fixed (4% formaldehyde, 10 min, RT) prior to analysis. Adipogenic differentiation was quantified using the ImageXPress Pico High Content Analyser (Molecular Devices, LLC). To assess 2D adipogenic differentiation, cells were stained with PBS solution containing Hoechst 34580 (1:5000; Sigma-Aldrich, 63,493) and BODIPY 493/503 (1:1000; Thermo Fisher, D3922) for 30min at RT. To identify positive cells, HCA software assigned lipid droplets stained with BODIPY to the nearest nuclei. Total BODIPY area or total lipid area was estimated per surface of a well.

#### Immunohistochemistry staining in 2D

To assess differentiation at a protein level the samples were blocked/permeabilized in blocking solution (PBS, 5% goat serum and 0.1% Triton X) at RT for 2 hrs. Permeabilized samples were incubated overnight at 4°C in PLIN1, CEBPɑ, PPARγ and ACACA primary antibodies (Cell signalling technology) at a dilution of 1:200 in blocking solution. After washing three times with buffer solution I (66 mM CaCl_2_ buffered with 10 mM HEPES), the fibres were incubated with donkey anti-rabbit Alexa 594 (1:250; R37119, Thermo Fisher) for 60 min at RT, followed by 15 min of Hoechst (1:5000) in PBS and three PBS washes. Samples were then imaged with confocal microscopy (TCS SP8, Leica Microsystems).

#### Lipid staining for adipogenic microfibres

For lipid visualisation, microfibres were stained overnight with Nile Red (a stain for neutral lipids) and Hoechst nuclei stain on days 7 and 28. To start, the microfibres were fixed using 4% paraformaldehyde in buffer solution I for 1hr at RT, and washed twice with buffer solution I. Once fixed, the microfibres were incubated overnight at 4°C in 1:500 Nile Red (N3013, Sigma Aldrich) and 1:625 Hoechst and washed again with buffer solution I. Finally, the microfibres were imaged by confocal microscopy using a 10/25× objective lens. For all confocal images, stacks were acquired using 2 μm Z-steps.

#### Immunofluorescence for adipogenic microfibres

For immunofluorescence staining for PLIN1 the samples were blocked/permeabilized in blocking solution (66 mM CaCl2, 10% goat serum and 0.1% Triton X) at RT for 2 hrs. Permeabilized microfibres were incubated overnight at 4°C in PLIN1 primary antibody (Cell signalling technology, 9349) at a dilution of 1:200 in blocking solution. After washing three times with buffer solution I, the fibres were incubated with donkey anti-rabbit Alexa 594 (1:250) for 90 min at RT, followed by three final washes. The samples were imaged as described above.

#### RT-qPCR

RNA was isolated using the E.Z.N.A Total RNA kit II (Omega Bio-tek, R6934). RNA was reverse transcribed using the iScript cDNA synthesis kit (Bio-Rad, 1708891) according to the manufacturer’s instructions. RT-qPCR was performed using iQ SYBR Green Supermix (Bio-Rad, 1708880) with primer pairs detailed in Table S2. 2^−ΔΔCt^ values for genes of interest were normalised to the average of three housekeeping genes (*UXT, RPL19,* and *RPLP0*). Each adipogenic marker value is normalised to its day 0 value. For better comparison within conditions, all conditions within one isolation were averaged and subsequently each condition was divided by the average of the same isolation. This was done to preserve the trend between isolations and overcome great isolation to isolation variations.

#### Glycerol assay

Media was refreshed every 3 or 4 days in the adipogenic microfibres culture, spent media was collected just prior to the media change at week 1 and 4. Glycerol was measured at these two time-points from three independent experiments on a metabolite analyser (CEDEX Bio Analyser; Roche).

#### RNA sequencing and analysis

SVCs were seeded in 2D at 3×10^4^ cells/cm^2^, proliferated for a day in SFGM and differentiated for 12 days in rDM. mRNA was extracted using the Dynabeads® mRNA DIRECT™ MicroKit (Thermo Fisher). Subsequently 1 ng of mRNA was reversed transcribed into cDNA using the Maxima H Minus Reverse Transcriptase (Thermo Fisher) and the primers from the PCR-cDNA Barcoding kit (SQK-PCB109, Oxford Nanopore Technologies). The cDNA was then barcoded using the same PCR-cDNA barcoding kit, which was then also used to finish the library prep. The libraries were sequenced using two R9.4.1 flowcells (Oxford Nanopore Technologies) on a GridION (Oxford Nanopore Technologies) for 48 hours.

Basecalling was performed using Guppy version 5.0.16 in the Super-accurate setting (minimal q-score threshold of 10) with automatic de-multiplexing and barcode removal.

The filtered reads were mapped to the bovine transcriptome (ARS-UCD1.2) using Minimap2^79^ with the default settings for Oxford Nanopore reads (-ax map-ont), and then subsequently quantified using Salmon.^80^

The data analysis was performed using DEseq2,^81^ with tximport^82^ for importing the quantified data from Salmon. Batch correction for the 3 separate batches of mRNA extraction and cDNA making was done using Combat-Seq from the SVA package.^83^^,^^84^ After differential gene expression analysis, gene set enrichment analysis for GO terms^85^^,^^86^ and WikiPathways^87^ was performed using the Piano R package^88^ using the following 7 methods: mean, median, sum, GSEA, PAGE, stouffer’s and tail Strength with 10,000 permutations each and a gene set size limit of min 10 and max 500. The median adjusted p Value across all methods is reported for each gene set as the final p Value. All steps were performed in-house, including the use of the Oxford nanopore and the data analysis.

#### Lipidomic analysis

Cultured fat samples (10–30 mg) were collected for lipidomic analysis. The samples were then sent to Lipometrix, the KU Leuven lipidomics core facility, to carry out the processing and analysis of lipids as described here. Lipids were extracted using a modified Bligh-Dyer protocol and analysed by hydrophilic interaction liquid chromatography mass spectrometry (HILIC LC-MS/MS).^89^ Lipid quantities were normalised to the amount of DNA present within the respective sample. For data analysis, peak integration was performed with MultiQuantTM (version 3.0.3). Lipid species signals were corrected for isotopic contributions (calculated with Python Molmass 2019.1.1) and quantified based on internal standard signals as per the Lipidomics Standards Initiative (LSI).

### Quantification and statistical analysis

Statistical analysis was performed using Prism 9.1.0 (GraphPad). For comparing two groups unpaired *t*-test was performed. Analysis of three or more groups was performed using one-way ANOVA with Bonferroni’s multiple comparisons test against indicated control(s). Analysis of three or more groups with two independent variables was performed using a two-way ANOVA with Bonferroni’s multiple comparison test against indicated control(s). Additional details on the statistical analysis of each experimental group can be found in the figure legends. Error bars in all figures represent the mean ± standard deviation (SD). N represents the number of donor animals. Technical replicates (multiple wells) were averaged, and statistical analysis was performed on biological replicates and replicates of individual experiments.

## Data Availability

RNA-seq data have been deposited at GEO (GEO:GSE206990) and are publicly available as of the date of publication. All the other data reported in this paper will be shared by the lead contact upon request.
