# Supplementary material for: A simplified and defined serum-free medium for cultivating fat across species
Source: iScience. 2022 Dec 17;26(1):105822. doi: 10.1016/j.isci.2022.105822 (PMC9830212; doi:10.1016/j.isci.2022.105822)
Supplement: Document S1. Figures S1–S7 and Tables S1–S3 [file mmc1.pdf]

iScience, Volume *26*

Supplemental information

A simplified and defined serum-free medium for cultivating fat across species

Rada Mitić, Federica Cantoni, Christoph S. Börlin, Mark J. Post, and Laura Jackisch

List of abbreviations and acronyms used in the paper

Abbreviation	Definition
3%FBS	Serum containing differentiation media (DMEM + 3% FBS)
-ctrl	No inducers (apart from insulin in DMAD), i.e. maintenance media
cDM (2D)	Control differentiation medium. Induction (4 days) –» Progression (4 days)
cDM (3D)	Control differentiation medium. Induction (4 days) –» Progression (24 days)
d	Days
DMAD	In-house developed Defined Media for Adipogenic Differentiation
Dex	Dexamethasone
FA	Fatty acid
HC/PR	Hydrocortisone and progesterone
I	Induction (i.e. rosiglitazone + insulin + dexamethasone +IBMX)
Ins	Insulin
IBMX	3-Isobutyl-1-methylxantine
M	Maintenance media (i.e., no inducers apart from insulin)
P	Progression media (i.e., rosiglitazone + insulin)
PUFA	Poly-unsaturated fatty acid
RA	Retinoic acid
Ros	Rosiglitazone
rDM (2D & 3D)	Reduced differentiation medium (rosiglitazone and insulin the whole time)
VFA	Volatile fatty acid
v	Vitamin

Gene abbreviation	Gene name
ACC	Acetyl-CoA carboxylase 1
ADIPOQ	Adiponectin
CIDEC	Cell death activator
PPARγ2 / PPARγ	Peroxisome proliferator-activated receptor gamma (two)
C/EBP	CCAAT enhancer binding proteins
SCD	Stearoyl-CoA desaturase
PLIN1	Perilipin 1
UCP-1	Uncoupling protein-1
ELOVL6	Elongation of very long chain fatty acids protein 6
APOE	Apolipoprotein E
COX6A1	Cytochrome c oxidase subunit 6A1

Reference	Basal media (+) FBS concentration	Days between media changes	Induction / Progression components: Insulin concentration	Rosiglitazone concentration	Dexamathasone concentration	IBMX concentration	Other DM components
doi.org/10.2508/ chikusan.69.439	DMEM:Ham-F12 (1:1), SF	10	10 µg/ml	-	-	-	vC, vB5, vH, vB7, Transferrin
doi:10.1016/ j.ygeno.2012.06.005	DMEM/F12 + 10% FBS	2	5 ng/ml	-	10 nM	-	vC, vB5, vH, T3, li-pids
doi.org/10.5713/ ajas.2013.13559	DMEM + 10% FBS	2	50 ng/ml	0 µM / 5 µM	250 nM	-	Octanoate, acetate
doi.org/10.3390 ijms151121401	DMEM + 5% FBS	2	5 µg/ml / 5 µg/ml	5 µM / 5 µM	0.25 µM	0.5 mM	-
doi.org/10.1007/ s11626-017-0205-7	DMEM + 10% FBS	21	10 µM / 10 µM	-	1 µM / 1 µM	0.5 mM / 0.5 mM	Indomethacine 200µM
doi:10.1017/ S1751731118000150	DMEM + 10% FBS	3	1 µg/ml / 1 µg/ml	-	0.1 µg/ml	27.8 µg/ml	-
doi.org/10.3168/ jds.2018-15626	DMEM/F12 + 10% FBS	2	5 µg/ml / 5 µg/ml	(Troglitazone) 5 µM / 5 µM	1 µM	0.5 mM	Acetate
doi:10.1111/ asj.12101	DMEM + 10% FBS	2	10 µg/ml / 5 µg/ml	-	1 µM	0.5 mM	RA
doi:10.1016/ j.cbd.2010.06.004	DMEM/F12+10%FBS	3	5 ng/ml / 5 ng/ml	-	10 nM / 10 nM	0.1 µM / 0.1 µM	vC, vH, vB5, lipids, T3
doi: 10.1159/000329254	DMEM/F12+10%FBS	2	2.5 µg/ml / 2.5 µg/ml	(Troglitazone) 5 µM / 5 µM	0.25 µM	0.5 mM	-
doi:10.1016/ j.abb.2019.108236	DMEM/F12 +10%FBS	2	167 nM / 167 nM	-	1 µM	0.5 mM	Hydrocortisone
doi:10.1007/s11745-013-3823-1	DMEM + 5% FBS	-	2.5 µg/ml / 2.5 µg/ml	(Troglitazone) 5 µM / 5 µM	0.25 µM	0.5 mM	Acetate
doi.org/10.17582/ journal.pjz/20190718150746	-	3	3 µg/ml / 3 µg/ml	1 mM	1 mM	0.5 mM	BMP4
doi:10.2527/ jas.2008-0860	DMEM	2	280 nM	(Troglitazone) 40 µM	0.25 mM	-	vB7, vB5, vC, lipid supplement, glucose

Table S1. Selection of different bovine adipogenic differentiation protocols. Related to Figure 1. The use of various traditional differentiation cocktails demonstrates the discrepancy between compounds in use and their concentrations.

Gene name	Primer sequence	
UXT	Fwd	5′-GAGCAGTCTCCTCACAGAGCTC
	Rev	5′-AGCAACATGTGGATATGGGCCT
RPL19	Fwd	5′-TCGAATGCCCGAGAAGGTAAC
	Rev	5′-CTGTGATACATGTGGCGGTC
RPLP0	Fwd	5′-GGCAGCATCTACAACCCTGA
	Rev	5′-CAGATGCGACGGTTGGGTAA
ADIPOQ	Fwd	5′-GGCTCTGATTCCACACCTGA
	Rev	5′-TGTTGTCCTCGCCATGACTG
CIDEC	Fwd	5′-TGCAGAGTAACCACTGCTGA
	Rev	5′-ACGCCAGCATCAGGGTATC
PPARγ	Fwd	5′-TGTCACAGTGTCTGCAAGGACC
	Rev	5′-ACGGAGCTGATCCCAAAGTTGG
SCD	Fwd	5′-GACCTAAGAGCCGAGAAGCTGG
	Rev	5′-ATCCCACAGATACCATGGCACG

Table S2. Primer sequences for RT-qPCR. Related to STAR Methods.

Media	Component	Concentration
Growth Medium (GM)	DMEM +	-
	Fetal Bovine Serum (FBS)	10%
	FGF2	2 ng/ml
	Penicillin/Streptomycin/Amphotericin (PSA)	1%
Serum-Free Growth Medium (SFGM)	DMEM/F12 +	-
	Chemically-defined FBS replacement (Kolkmann et al., 2022)	1%
	PSA	1%
3% FBS Differentiation Medium (3%FBS)	DMEM +	-
	FBS	3%
	PSA	1%
	± Insulin	10 µg/ml
	± Rosiglitazone	5 µM
	± IBMX	0.5 mM
	± Dexamethasone	1 µM
Defined Medium for Adipogenic Differentiation (DMAD)	DMEM/F-12 +	-
	PSA	1%
	HEPES	4.9 mM
	Lipid concentrate	0.1%
	Putrescine	57 µM
	± Progesterone	17.8 nM
	± Hydrocortisone	25 nM
	Calcium Chloride	1 mM
	L-Ascorbic acid 2-phosphate	227 µM
	Glucose	17 mM
	FGF2	2 ng/ml
	EGF1	2 ng/ml
	BMP4	10 ng/ml
	± Insulin	10 µg/ml
	± Rosiglitazone	5 µM
	± IBMX	0.5 mM
	± Dexamethasone	1 µM

Table S3. Composition of proliferation and differentiation media used in this study. Related to STAR Methods.

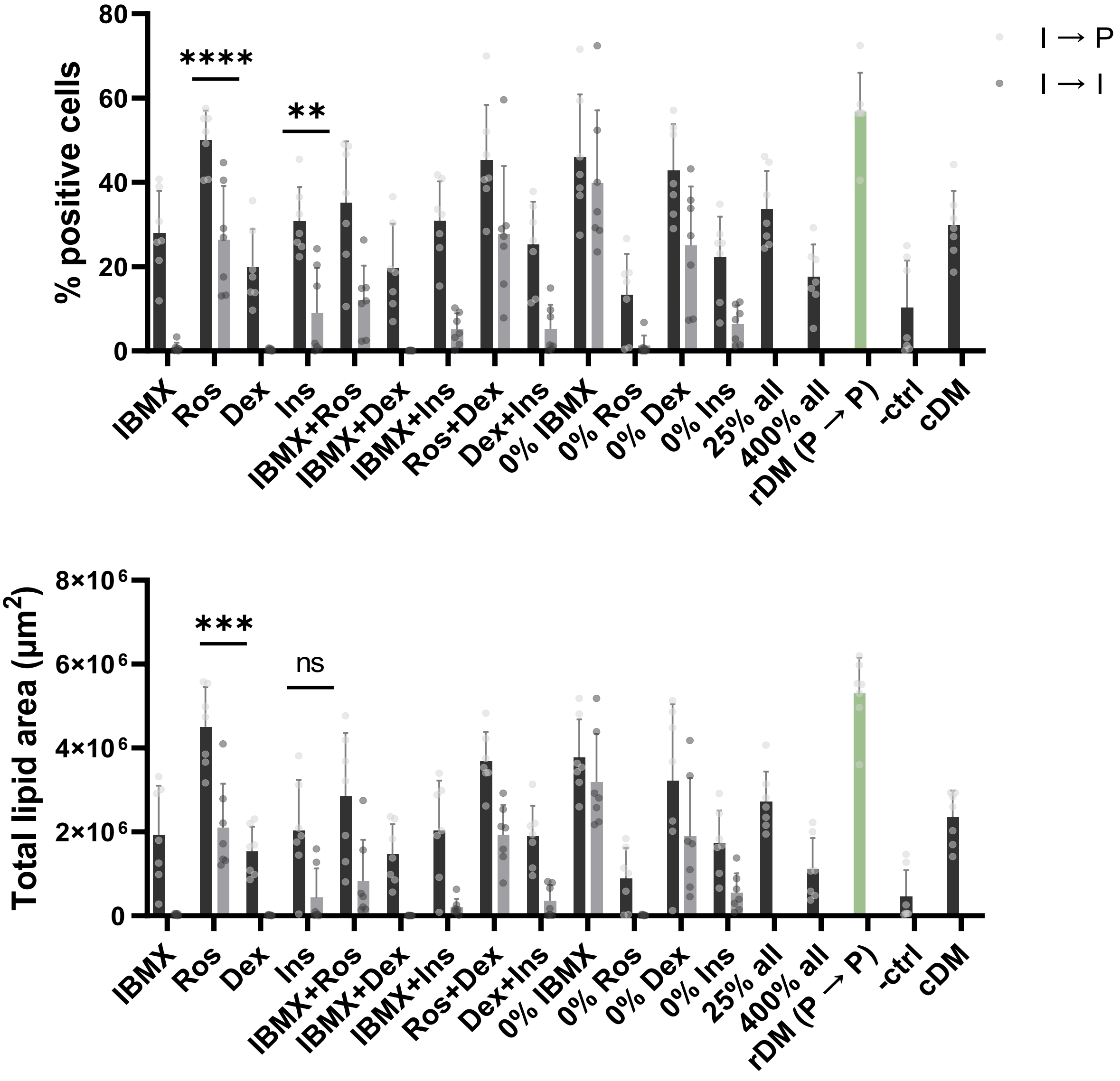

Figure S1. Quantitative assessment of adipogenic differentiation with different inducer combinations at day 8. Related to Figure 3.

Different combinations of differentiation medium compounds were used to investigate the necessity of adipogenic inducers. I –» P indicates compounds present only during first media change, followed by progression in the second media change. Alternatively, I –» I indicates compounds/combinations maintained throughout 8 days of differentiation (inducers were supplemented in first and second media changes). Data are represented as mean ± SD; the error bars represent the SD of 4 independent experiments using 4 donors. One or two-way ANOVA; NS, not significant; **P < 0.01; ***P < 0.001 and ****P < 0.0001.

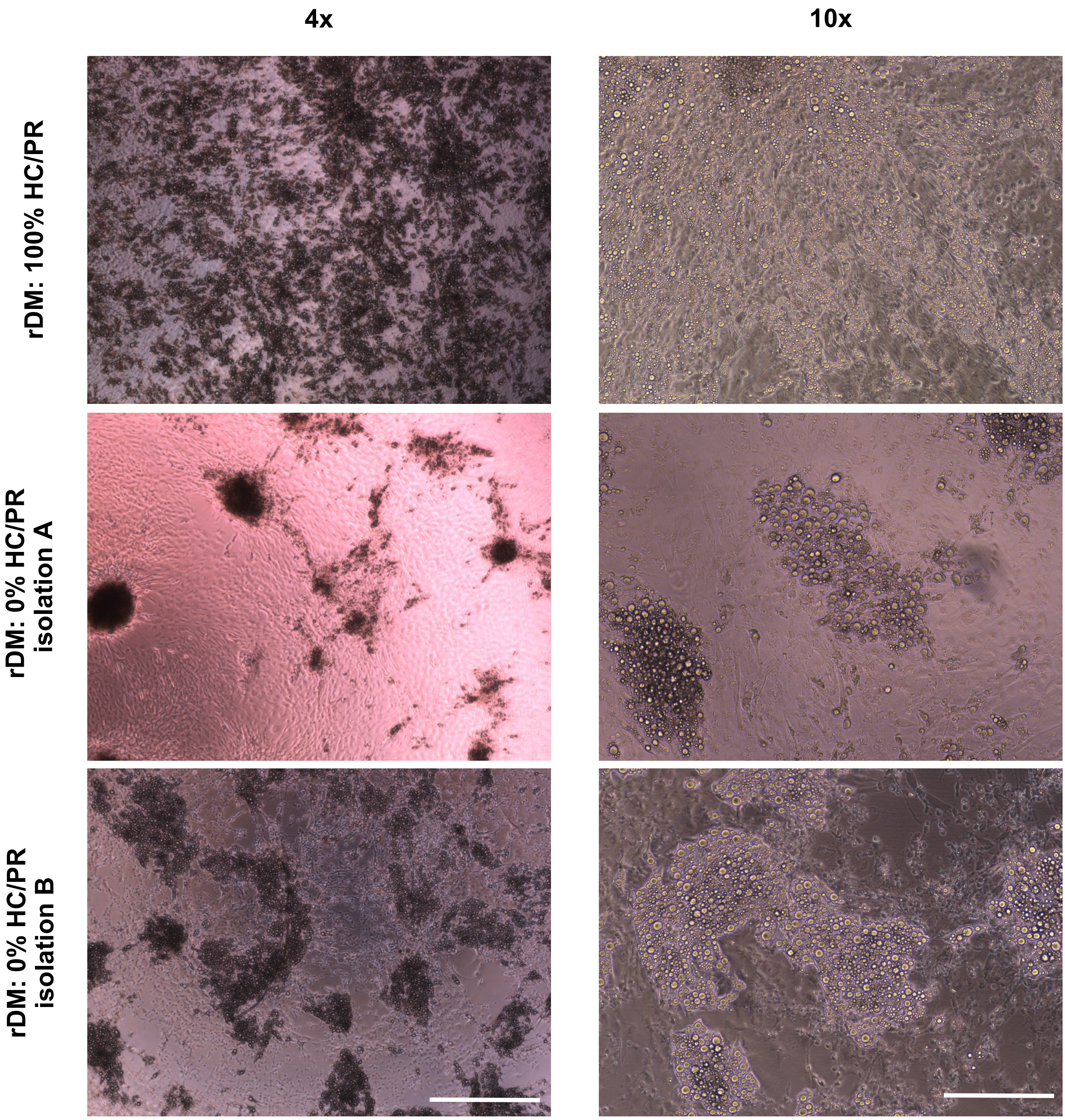

Figure S2. Excluding all the GC receptor activating molecules (HC/PR) resulted in islands of differentiation and cell death in specific isolations. Related to Figure 3d.
SVC were proliferated in SFGM and differentiated with rDM, or rDM without HC/PR. Pictures were taken at day 12, before harvesting mRNA from two independent donors. Scale bars 700 µm (4x magnification) or 300 µm (10x magnification).

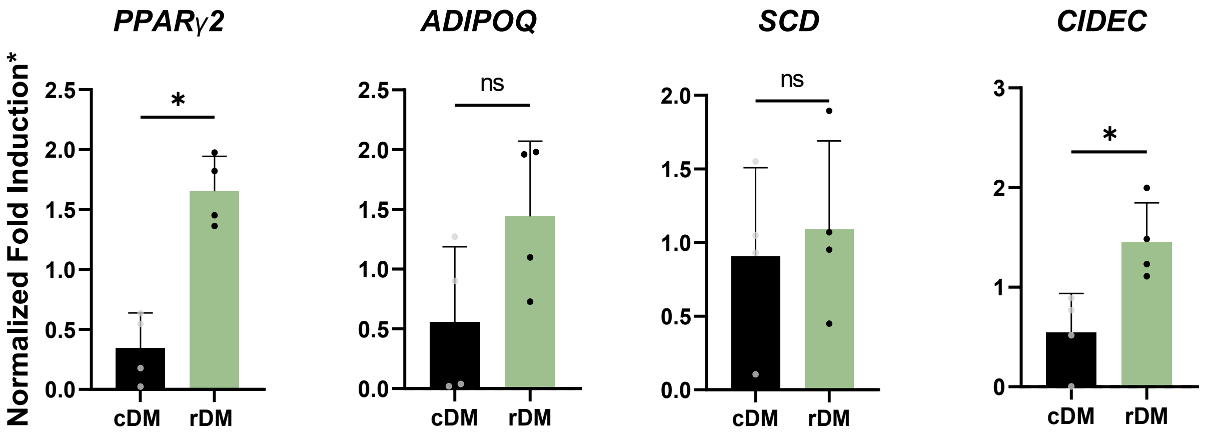

Figure S3. Cells differentiated with rDM present higher expression of two adipogenic markers in 3% FBS. Related to Figure 4a.
Mean normalized gene expression fold changes 12 days after induction of differentiation determined by qPCR. All conditions were normalised to a chosen set of reference genes (*UXT, RPLP, L19)* and to day 0 control (not shown); *mean $2^{-\Delta\Delta Ct}$ values of each condition were divided by the average of same isolation to improve comparability between conditions. Data are represented as mean ± SD; the error bars represent the SD of 4 independent experiments using 4 donors. One-way ANOVA; NS, not significant; *P < 0.05.

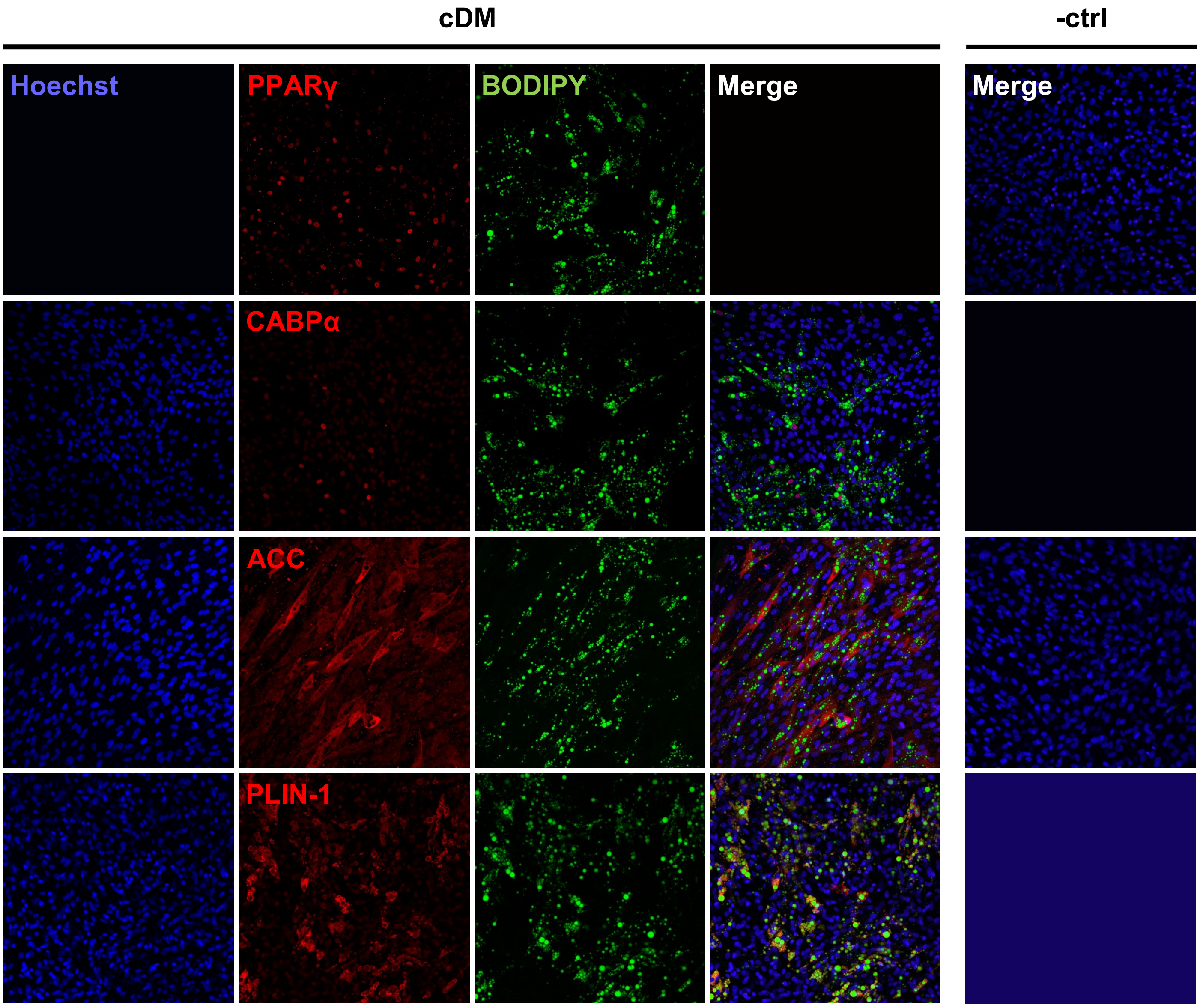

Figure S4. Immunohistochemical analysis of bovine SVC. Related to Figure 4b.
Cells were differentiated with control differentiation medium (cDM; I –» P) or negative control (no inducers, except insulin) for 8 days. Scale bar, 100 µm.

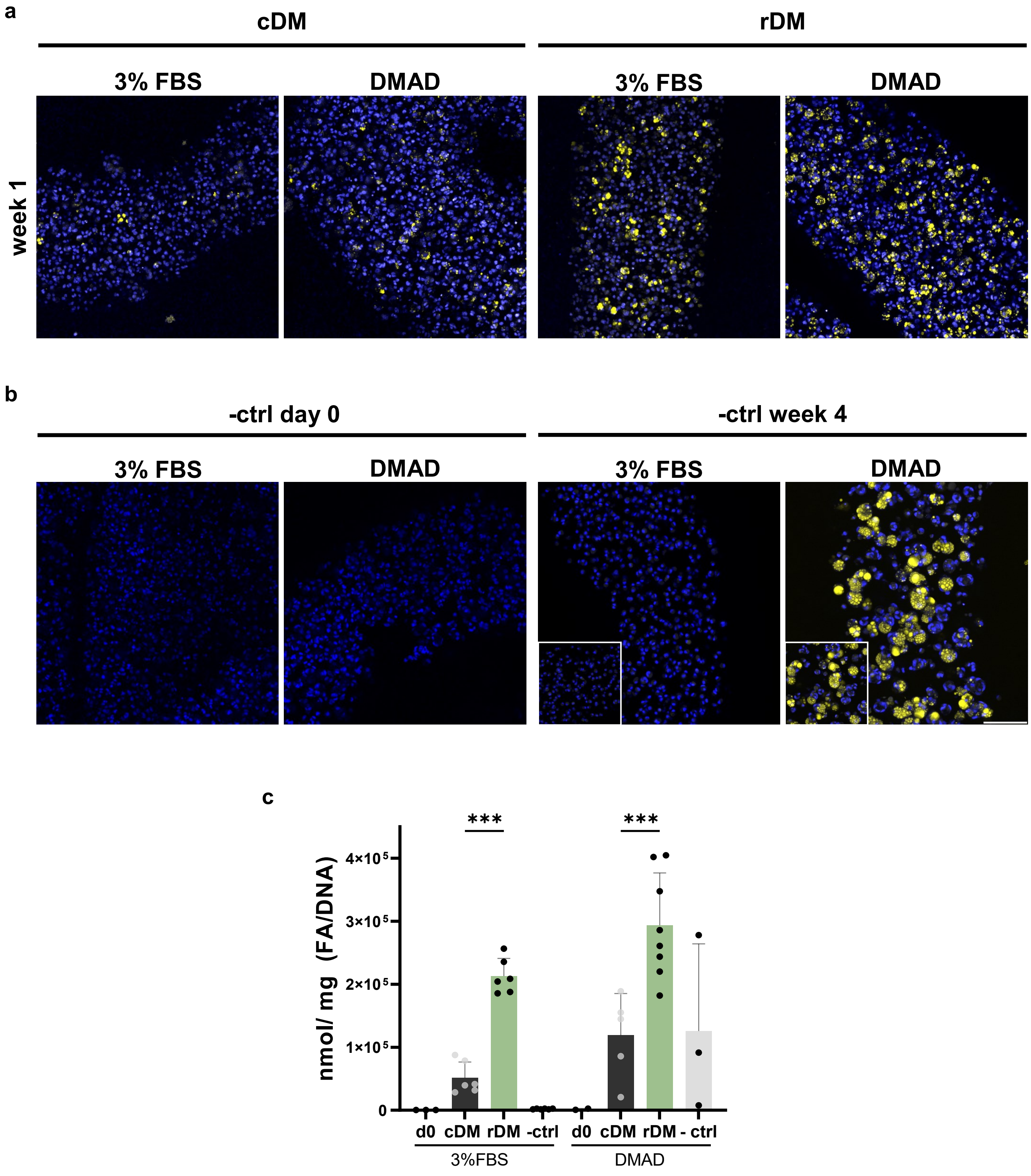

Figure S5. At week one rDM already outperforms cDM. Related to Figure 5.
Bovine SVC were differentiated in alginate fibres with 3%FBS or DMAD with the standard differentiation cocktail (cDM), rDM or negative control (-ctrl DMAD contains insulin and HC/PR). The culture was stopped at weeks 1 and 4. **a,** Representative images of rDM and cDM after one week of differentiation in 3%FBS or DMAD. **b,** Negative control differentiation at day 0 and week 4. **c,** Fatty acid quantity within triglycerides (TG) normalised to the amount of DNA. Data are represented as mean ± SD; the error bars represent the SD of 3-5 independent experiments using 2-5 donors. Statistical analyses were performed using a one-way ANOVA; ***P < 0.001. Scale bar, 100 µm.

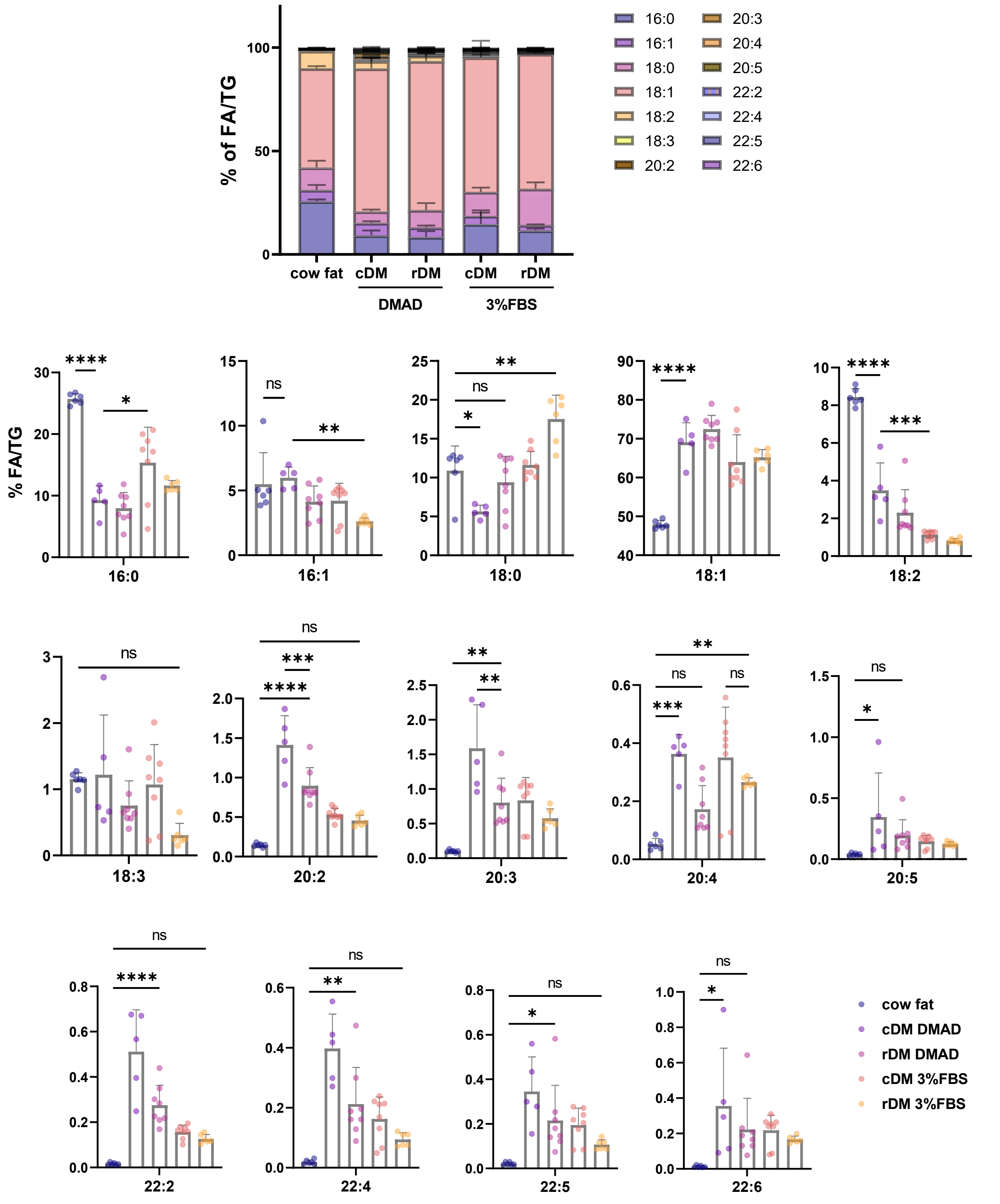

Figure S6. Lipidomic assessment of fatty acid percentage within triglycerides. Related to Figure 5e. Bovine SVC were differentiated in alginate fibres with 3%FBS or DMAD with standard differentiation cocktail (ctrl) or rDM for 28 days. Fibres were collected and analysed at week 4. Data are represented as mean ± SD; the error bars represent the SD of 3 to 5 independent experiments, n=5 donors (cDM DMAD); n=8 donors (rDM DMAD and cDM 3% FBS); n=6 donors (bovine fat and rDM 3% FBS). Statistical analyses were performed using a one-way ANOVA; NS, not significant; *P < 0.05; **P < 0.01; ***P < 0.001; and ****P < 0.0001.

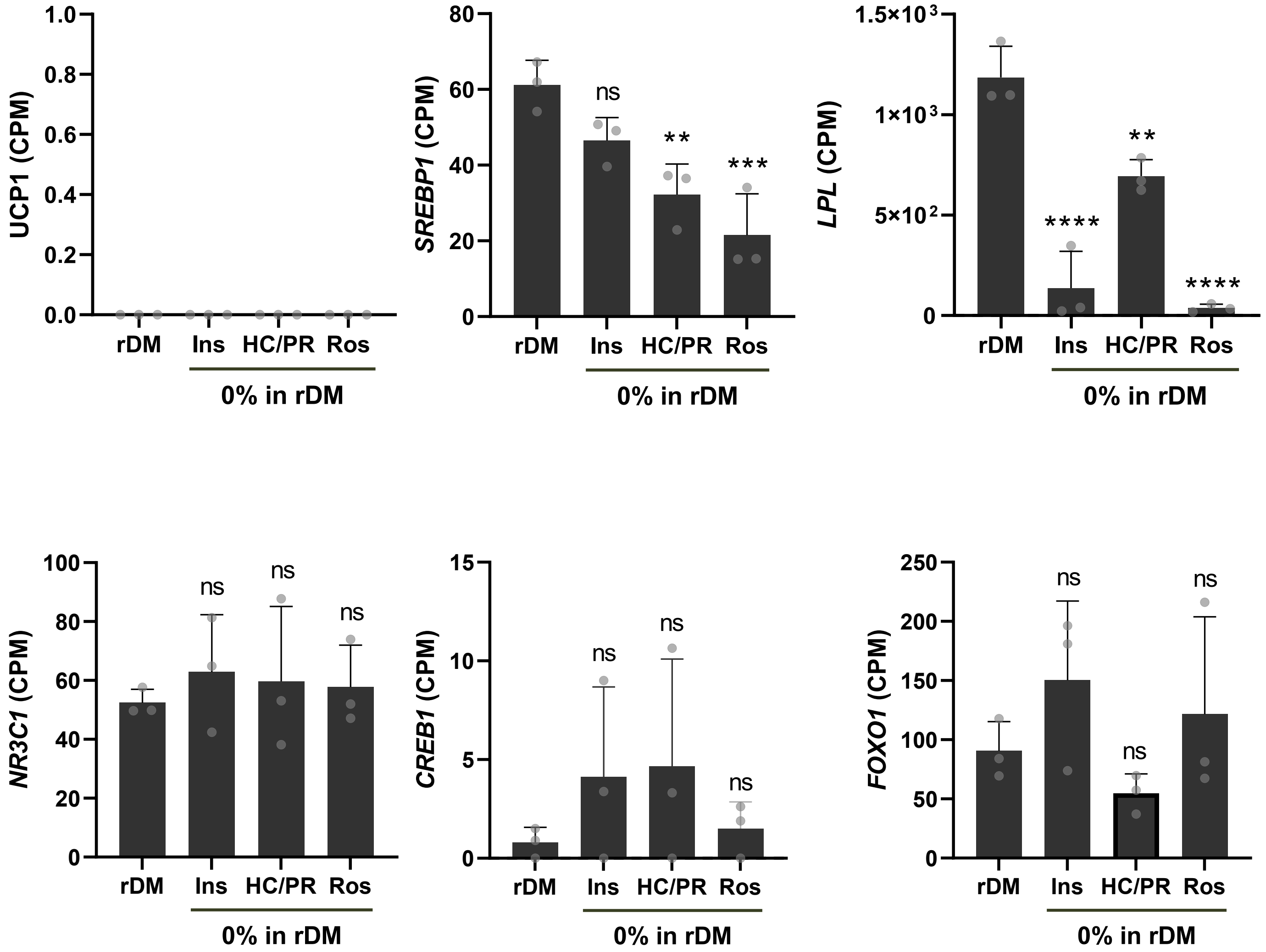

Figure S7. Transcriptomic analysis demonstrates impact of insulin, rosiglitazone and glucocorticoid receptor binding molecules on adipogenic markers. Related to Figure 6c. SVC were proliferated in SFGM and differentiated with rDM, or rDM without insulin, rosiglitazone or HC/PR. mRNA was harvested at day 12. Selection of differentially expressed gene: Uncoupling protein 1 (*UCP1*), a major marker of brown adipogenesis; Sterol regulatory element binding protein-1 (*SREBP-1*); Lipoprotein lipase (*LPL*) tryglyceride metabolism; Glucocorticoid receptor (NR3C1); Cyclic AMP-responsive element-binding protein 1 (*CREB*); and Forkhead box protein O1 (*FOXO1*). Data are represented as mean ± SD; the error bars represent the SD of 3 independent experiments using 3 donors. Statistical analyses and comparisons were performed using a two-way ANOVA; NS, not significant; **P < 0.01; ***P < 0.001; and ****P < 0.0001.
